# Ethical reflections on the COVID-19 pandemic in the global seafood industry: navigating diverse scales and contexts of marine values and identities

**DOI:** 10.1007/s40152-021-00247-w

**Published:** 2021-10-20

**Authors:** Mimi E. Lam

**Affiliations:** Centre for the Study of the Sciences and the Humanities, University of Bergen, P.O. Box 7805, N-5020 Bergen, Norway

**Keywords:** Ethical development, Ethical governance, Identities, Seafood value chains, Trade-offs, Values

## Abstract

The global crisis instantiated by the COVID-19 pandemic opens a unique governance window to transform the sustainability, resilience, and ethics of the global seafood industry. Simultaneously crippling public health, civil liberties, and national economies, the global pandemic has exposed the diverse values and identities of actors upon which global food systems pivot, as well as their interconnectivity with other economic sectors and spheres of human activity. In the wake of COVID-19, ethics offers a timely conceptual reframing and methodological approach to navigate these diverse values and identities and to reconcile their ensuing policy trade-offs and conflicts. Values and identities denote complex concepts and realities, characterized by plurality, fluidity and dynamics, ambiguity, and implicitness, which often hamper responsive policy-setting and effective governance. Rather than adopt a static characterization of specific value or identity types, I introduce a novel hierarchical conceptualization of values and identities made salient by scale and context. I illustrate how salient values and identities emerge at multiple scales through three seafood COVID-19 contextual examples in India, Canada, and New Zealand, where diverse seafood actors interact within local, domestic (regional/national), and global seafood value chains, respectively. These examples highlight the differential values and identities, and hence differential vulnerabilities, resilience, and impacts on seafood actors with the COVID-19 pandemic, which necessitate differentiated policy interventions if they are to be responsive to those affected. An ethical governance framework that integrates diverse marine values and identities, buttressed by concrete deliberation and decision-support protocols and tools, can transform the *modus operandi* of global seafood systems toward both sustainable *and* ethical development.

## Introduction

The global coronavirus disease 2019 (COVID-19) pandemic has instantiated an unprecedented public health crisis that has severely disrupted worldwide social mobility and economic sectors. Policy interventions in response to the pandemic have extended government powers in liberal democracies, restricting individual freedoms, while in authoritarian regimes, power has been seized, media censored, and citizens tracked. Fear of contagion and increasing frustration among citizens with COVID-19 policy restrictions have heightened inter-group differentiation and politicized the pandemic, manifest in rampant racism, discrimination, and conflict (Lam 2021). Government policy responses to the COVID-19 pandemic also have ignited debates about which societal values to prioritize, notably among public health, civil liberties, and the economy (ibid.). The pandemic has exacerbated and exposed value conflicts, accentuating the need to reform management and governance systems so that they reflect and re-prioritize societal values, both market and non-market. It also has accentuated the interconnections among nations, sectors, and varied spheres of human life (e.g., health, the economy, civil society, politics, law, and the environment) that specific policy responses and governance frameworks must account for to be effective within today’s highly complex, diverse, and interconnected world.

In the global seafood industry and other economic sectors, the COVID-19 pandemic has exposed and intensified societal vulnerabilities and value conflicts associated with market value priorities. Governments, markets, and civil societies have differential resilience to cope with the pandemic, accentuating the differential vulnerabilities of, and impacts on particular social actors and groups within and across multiple scales and contexts. Seafood value chains, for example, consist of diverse seafood actors representing plural values and identities, including producers, processors, distributors, wholesalers, retailers, and consumers, in varied global contexts (Lam 2016, 2019a, b). Differential vulnerabilities and resilience of seafood actors and value chains to COVID-19 have highlighted disparities in terms of COVID-19 impacts across individual fishworkers, social and sectoral communities, and national societies and economies. COVID-19 impacts within the global seafood industry vary with scale and context according to, *inter alia*, pandemic severity, policy measures, and resilience of actors, species, fleets, value chains, and nations. The ongoing ripple effects of the global pandemic to public health, social mobility, and national economies afford a unique opportunity to reflect on, and potentially reconfigure public policies related not only to COVID-19, but also to the global seafood industry (Lam 2021).

To address the differential vulnerabilities of and impacts on diverse seafood actors within the global seafood system, as accentuated by the COVID-19 pandemic, I offer an ethical perspective with broad conceptual, methodological, procedural, and consequential governance implications:

1. Ethics as a *conceptual framework or lens* for global seafood governance post-COVID-19, which focuses on the inherent plurality of values and identities in the industry and society and the ensuing value conflicts and trade-offs that policies and governance must navigate;
2. A transformative *methodological approach* to governance that is ethically grounded, with concrete ethical procedural guidelines and participatory tools for deliberation and decision-support that would foster more sustainable and ethical seafood outcomes;
3. *Deliberative and decision*-*support protocols* that embrace the plurality of values and identities in the marine sector to avoid the adverse consequences of undifferentiated policy and governance interventions, particularly on marginalized individuals, groups, and populations (e.g., women, indigenous and rural communities, and ethnic minorities); and
4. Illustrative contextual examples that extrapolate from values research to link COVID-19 *consequences* in three places (India, Canada, and New Zealand) to broader trends in the marine realm, namely, *policy and governance implications* of explicating the typically implict values and identities of actors at multiple scales in diverse seafood value chains.

Supplying food, including seafood, has remained an essential service throughout the pandemic. Global food supply chains have shown resilience (Rubinstein 2020), with some countries, like New Zealand, re-configuring food systems to enhance their domestic resilience against external disruptions (Kaiser et al. 2021). Resilient food systems often have flexible supply chains, such as alternative seafood networks, which add functional diversity that can handle systemic shocks (Stoll et al. 2021). The global food system is designed for maximum (economic) efficiency and relies on the stability and predictability of trade in food commodities: food stocks are managed in “just-in-time” systems, with food inputs delivered when needed to avoid costly storage and loss of freshness in large warehouses (Wong 2020). With COVID-19 disruptions to social mobility and transport, food stopped arriving “just in time,” with the labor-intense nodes of the food chain hardest hit, i.e., food production and harvesting, processing, and stocking of store shelves (ibid.). High infection rates in meat-processing facilities (Corkery and Yaffe-Bellany 2020) and on factory fishing vessels (Herz 2020) exposed the vulnerability and working conditions of food laborers. Technological responses to COVID-19 include greater mechanization to reduce dependence on food laborers and improved communication, transparency, and trust to enhance flexibility in food chains (Wong 2020). Some producers are selling directly to consumers in direct marketing solutions to shorten supply chains, while processing innovations are extending the shelf life of perishable foodstuffs (ibid.). Ultimately, adding diversity along the entire food chain, with hybrid local and global solutions of getting food to market, will enhance the resilience, viability, and health of integrated food systems to future shocks, thus promoting their overall sustainability and ethics (Lam and Pitcher 2012; Lam 2016).

This paper focuses an ethical lens on the differential COVID-19 vulnerabilities of and impacts on diverse social actors within the global seafood industry. These differential vulnerabilities and impacts are contextualized within an ethical conceptual and methodological framework that is premised on the recognition and reconciliation of value and identity plurality within society. As background, four general characteristics are identified of values and identities that complicate responsive policy-setting and effective governance, namely plurality, fluidity and dynamics, ambiguity, and implicitness. Next, three COVID-19 seafood contextual examples—in India, Canada, and New Zealand—illustrate how the pandemic has exposed salient values and identities at multiple scales (viz*.*, individual, social, and cultural) within the global seafood industry, spanning local, domestic (regional/national), and global value chains, respectively. Concrete deliberation and decision-support protocols and tools are described that can help navigate diverse scales and contexts of marine values and identities, resolve their ensuing conflicts, and reconcile policy trade-offs among competing objectives related to the global seas. Such an ethical governance framework has the potential to transform global seafood systems post-COVID-19 by enabling and facilitating not only sustainable, but also ethical development.

## Ethical contextualization of global seafood governance

Environmental resource problems have non-unique problem-framings and solutions, as individuals and stakeholder groups not only value natural systems differently, but also are differentially affected by policies, which can lead to conflicts. This philosophical perspective (Ariansen 1992) underscores the plurality of values that characterizes wicked policy problems (Rittel and Webber 1973; Jentoft and Chuenpagdee 2009; Lam et al. 2019) and post-normal science problems at the science-policy interface (Funtowicz and Ravetz 1990, 1993; Ainscough et al. 2018; Lam et al. 2019; Kaiser et al. 2021). Differential valuations of nature by diverse human actors and their different values and identities influencing their behaviors lead to complexity in governance of human-nature activities. Recognizing that conflicting values and identities are fundamental to seafood governance problems constitutes an important philosophical insight and addition to the governance literature, namely, its contextualization within an ethical framework.

Policy problems related to the global oceans, such as how to manage and to allocate marine resources, have both scientific and ethical dimensions, often manifest in resource conflicts and trade-offs. Scientific approaches to marine resource management are well developed (see, e.g., Hilborn et al. 2020), but ethical approaches to identify diverse marine values and identities, to evaluate policy trade-offs, and to reconcile conflicts are still nascent (but see Lam 2016, 2019a, b; Lam et al. 2019; Kaiser et al. 2021). Resource management goals are ultimately statements of values (Grumbine 1994), such as ecological, socio-cultural, and economic values, but they are typically only implicit in management frameworks (cf. Lam et al. 2019; Loring and Hinzman 2018), despite that specific value-based outcomes are selected in management and policy decisions. Meanwhile, identities, characterized by, e.g., nationality, ethnicity, race, religion, sectorality, socio-economic class, and gender, can infuse policy negotiations and political rhetoric with “identity politics” that unite members of in-groups and divide out-groups (Fukuyama 2018). Different actors (as individuals and stakeholder groups) often will frame problems and identify management goals and solutions differently, depending on their particular values and identities, which can lead to tensions and conflicts. Resolving such tensions and conflicts would benefit from the elucidation of salient values and identities and deliberative reconciliation of trade-offs. This is the goal of seafood ethics, defined as the empirical (descriptive and evaluative) study and normative reflection of values, value-based trade-offs, and ethical dilemmas of stakeholders and citizens interacting across diverse seafood value chains^1^ (Lam 2019a, b). By elucidating both facts and values and how they are often intertwined in science and policy, seafood ethics offers a guiding decision-analytic framework for sustainable and ethical seafood governance (ibid.).

The literature on values and identities is vast and contested, so here, I will not attempt a summary or even an unbiased account of these disciplinary perspectives (see, e.g., Brosch and Sanders 2016, for a broad review of value perspectives). Instead, I ground my contribution in a transdisciplinary perspective of values and identities resonant with conceptual insights and empirical analyses from diverse fields, for example: sustainability science (Kenter et al. 2019); environmental science (Dietz et al. 2005, Dietz 2013); political science (Fukuyama 2018); evolutionary biology (Graham et al. 2013); ecological economics (Kenter 2016; Kenter et al. 2015, 2016); social psychology (Schwartz 1992, 1994; Turner et al. 1994; Tsirogianni and Gaskell 2011); sociology (Hitlin and Piliavin 2004) ; anthropology (Graeber 2001); geography (Cheng et al. 2003); and philosophy (Tappolet and Rossi 2016). My analysis seeks to identify general characteristics of values and identities, rather than to adopt a static characterization of specific value or identity types that lack universal consensus and flexibility. I embed my analysis of values and identities within an ethical contextualization, offering deliberative and decision-support protocols and tools that can be used in concert with scientific approaches to analyze and resolve problems at the science-society-policy nexus. Such a coherent ethical and scientific decision-analytic framework can help to identify relevant values; to evaluate management objectives, criteria, and scenarios; and to reconcile trade-offs through inclusive deliberation, rational analysis, and transparent decision-making. Focusing an ethical lens on diverse values and identities in the global seafood industry made salient by the COVID-19 pandemic exposes not only the adverse impacts of policies that do not recognize or differentiate marine values and identities, but also the transformative possibilities for sustainable and ethical governance of policies that do.

Values are concepts or beliefs about desirable end states or behaviors that transcend specific situations, guide selection or evaluation of behavior and events, and are ordered by relative importance (Schwartz and Bilsky 1987). As complex ideals of what is preferable and desirable, values are recognized to define or direct goals, frame attitudes, and provide standards against which human behavior is judged (Leiserowitz et al. 2006). They serve as reference points for evaluating something as positive or negative, have both rational and emotional dimensions, and give long-term orientation and motivation for action (modified from Kaiser 2015). Values are extensively studied by multiple disciplines but lack a coherent theory (Brosch and Sander 2016). Different value typologies exist (e.g., Inglehart 1971; Schwartz 1992; Graham et al. 2013; Chan et al. 2016; Rabinowicz and Rønnow-Rasmussen 2016), but here, I focus on their contextuality (Tsirogianni 2009; Tsirogianni and Gaskell 2011) and scale (Kenter et al. 2019; van Riper et al. 2018). Salient values are often revealed by tension or conflict, such as the current pandemic, and the values that influence decisions and behaviors can vary with context and scale.

I conceptualize values hierarchically by the relevant scale of human organization, namely individual, social, and cultural. Individual values operate at the level of the individual, while both social and cultural values operate within and across groups or aggregates of individuals, that is, among disparate communities and societies, respectively. These latter shared values, however, are not simply the sum of individual values, but rather represent emergent properties at higher levels of human organization through internalization (scaling up of individual values to group values) and socialization (scaling down of group values to individual values) processes and the consequential impacts of individual behaviors on groups (see also Kenter et al. 2015, 2019). My hierarchical conceptualization of values deviates slightly from the multi-level value description of Kenter et al. (2015, 2016, 2019) and more significantly from van Riper et al. (2018), with parallels in multi-level governance (Bache and Flinders 2004; Saito-Jense 2015). Kenter et al. (2015, 2016, 2019) distinguish social or shared values from individual values as encompassing communal, group, cultural, and societal values. Communal values are described as “values held in common by members of a community (e.g., geographic, faith/belief-based, community of practice or interest), including shared principles and virtues as well as a shared sense of what is worthwhile and meaningful,” while group values represent, more restrictively, “values expressed by a group as a whole (e.g., through consensus or majority vote, or more informally), in some kind of valuation setting” (Kenter et al. 2015, p. 88). Cultural and societal values are distinguished as “culturally shared principles and virtues as well as a shared sense of what is worthwhile and meaningful” (ibid.). Cultural values are described as “grounded in the cultural heritage and practices of a society and pervasively reside within societal institutions,” while societal values are the multiple sets of potentially overlapping cultural values of a heterogenous society (ibid.). In contrast, van Riper et al. (2018, p. 2) differentiate individual, cultural, and assigned values, namely: individual values are “fundamental, guiding principles in life”; cultural values are “guiding worldviews—or ‘ways of life’—that define a society and encompass the dominant normative, attitudinal, and behavioral patterns that exist within and between collectives”; and assigned values are “the social aggregations of beliefs.” I agree with Kenter et al. (2016, p. 197) that the held-assigned dichotomization of values is “incomplete and ambiguous,” so I avoid it, but I deviate from Kenter et al. to differentiate between shared and social values. I conceptualize social values as values shared by individual members of a group at the community level and cultural values as sets of values shared by individuals and communities at the societal level.

Identities are intricately linked with values: values shape identities and identities shape values in complex, dynamic, coupled processes that cross scales and vary with context. “Personal identity refers to self-categories that define the individual as a unique person in terms of his or her individual differences from other (in-group) persons” (Turner et al. 1994, p. 454). That is, an individual’s personal identity centres on one’s uniqueness from all others and stems from one’s values, beliefs, emotions, personality traits, attitudes, etc. and relationships with others to define one’s sense of self. Social identity, meanwhile, refers to “social categorizations of self and others, self-categories that define the individual in terms of his or her shared similarities with members of certain social categories in contrast to other social categories” (ibid.). That is, social identity extends notions of personal identity to social groups, whereby an individual views her- or himself as a member of a group to which she or he belongs, as distinct from other groups (Tajfel and Turner 1986). Social identity thus refers to the “shared social categorical self” that identifies with social groups as a result of a developed sense of belonging to those groups, leading to “us” vs*.* “them” and “in-group” vs. “out-group” distinctions (Turner et al. 1994, p. 454). These social groups or identities can be defined by diverse singular or aggregated characteristics, such as gender, socio-economic class, and occupation, which its members have in common. Cultural identity consists of a broader set of values, beliefs, meanings, and customs shared with other members of one’s cultural group, characterized by, for example, religion, race, ethnicity, and nationality. These are not rigid, but rather loose categorizations representing different scales of identities that we construct to identify ourselves in relation to others (individuals and groups).

## Four general characteristics of values and identities

Values and identities are often invoked in political rhetoric to unite or divide, as witnessed during the COVID-19 pandemic (Lam 2021). But despite their policy and political salience, values and identities are not well understood or empirically studied in fisheries (or other disciplines). Fisheries science typically concentrates on the dynamics of fish populations to estimate the biomasses of target species to inform management and policy decisions regarding the harvest or catch quotas to be set for each fishing season. It biases that which can be quantified. Quotas are then divided up among fishing nations, sectors, gear types, etc., in complex political negotiation processes. The values and identities of actors along seafood value chains, be they fishers, processors, distributors, retailers, or consumers, typically are not considered in resource management or decision-making frameworks, which tend to rely heavily on resource ecology and economics. This prioritization of quantitative data and market values reflects an implicit bias in fisheries science and policy that has been exposed by the COVID-19 pandemic. Exploration of the full suite of values of different seafood actors, including non-market values, with methodologies incorporating qualitative data is needed to capture the diverse dimensions of fishery problems. Explicit consideration and understanding of the complexity of values and identities of social and scientific actors in fisheries is complicated by their plurality, fluidity and dynamics, ambiguity, and implicitness. These hamper responsive policy-setting and effective governance, but efforts are underway^2^ to articulate a science of values and identities in political and policy processes.

### Plurality

Individuals, communities, and societies hold plural established values and identities simultaneously that can align or conflict, which can then amplify or dilute actors’ behaviors or create tensions in different contexts and at different scales. For individuals, value priorities can vary with different roles and identities across life domains or “behavioral spheres,” such as familial, occupational, recreational, and political spheres or contexts (Kluckhohn and Strodtbeck 1961; Tsirogianni 2009; Tsirogianni and Gaskell 2011). Also, individuals may belong to multiple social groups and hence have multiple social values and group identities beyond their personal values and identities, which can vary in importance and degree of accessibility with situational context (Turner et al. 1994; Hogg and Reid 2006). Multiple group identities also can exist within communities and societies, such that across and within heterogeneous groups, the directive nature of values or value orientations (Kluckhohn and Strodtbeck 1961) and work values or organizational cultures (Hofstede 1980) can vary. Group identities can encompass national, cultural, ethnic, racial, gender, age, professional, religious, and sexual orientation identities, all of which can contribute to one’s political identity. In fisheries, for example, behavioral identities have been used to distinguish fishers by their fishing styles, which account not only for how, but also why they fish as they do, based on specific social-ecological contexts and individual choices (Boonstra and Hentati-Sundberg 2016). Thus, a plurality of relatively stable values and identities can exist at multiple scales (e.g., individual, community, and societal), influenced by different contexts (e.g., behavioral, social, economic, cultural, historical, political, and institutional). Recognizing this plurality of established values and identities across scales and contexts, and unraveling their origins, dynamics, and consequences, can nuance understanding of the behaviors of seafood actors and their differential vulnerabilities to seafood policies.

### Fluidity and dynamics

Related to the plurality of established or relatively stable values and identities is their fluidity across, and even within contexts and their dynamics over time. Their context-dependence and temporal dynamics result in shifting value and identity profiles, priorities, and salience. Both Schwartz’s theory of values (Schwartz 1992, 1994) and moral foundations theory (Graham et al. 2013) are based upon universal or fundamental human values, which do not capture how values may vary with context, time, and individual. Individuals belong simultaneously to various groups associated with multiple established social identities and which specific identity dominates in an individual’s self-conceptualization or categorization of others can vary with situational context. New identities can also emerge and coalesce with pre-existing ones. For example, the COVID-19 pandemic is emerging salient viral identities and instantiating multiple, fluid behavioral identities that are being hijacked by existing social and political identities, with ineffective outcomes and unintended consequences for undifferentiated policy interventions (Lam 2021). Similarly, the Rwandan genocide emerged from an economic class division between the Hutus (majority who farmed crops) and the Tutsis (minority elites who tended livestock) that escalated to an ethnic distinction, with dire humanitarian consequences (Beauchamp 2014). The tragic story of Sister Gertrude—a Christian, Hutu, and nun—illustrates how identities one assigns to oneself and to others can fluidly shift, even within a given context and time: on 6 May 1994 in Sovu, Rwanda, Sister Gertrude, identifying as a Hutu, denied protection to the Tutsi from the Hutu militia in the convent where she was mother superior, but identifying also as a nun, she protected the nuns who were Tutsi (Berreby 2005). In fisheries, values and identities are often ambiguous and implicit (see below), being prioritized by individuals depending upon the context, which may or may not reflect the divisive cultural identities among conflicting stakeholder groups (Lam et al. 2019). In the political sphere, politicians often use rhetoric to stimulate particular values and identities to gain political advantage or to make others invisible if suited to their interests.

### Ambiguity

Despite their centrality in orienting human behavior and activities (Brosch and Sander 2016), values are often ambiguous, varying with individual interpretations and specific contexts. For example, freedom has both positive and negative interpretations: freedom to control oneself and realize one’s potential; and freedom from control and interference by others, respectively (Berlin 1969). Freedom was invoked by fishers on opposing sides of a conflict in Canada to support divergent views: freedom from external market competition, with the aid of governmental regulation, to control their fishing activities and operations; and freedom from governmental interference in the marketplace (Johnson et al. 2019). In another Canadian fishery, two conflicting stakeholder groups, a predominantly indigenous community and the herring industry, prioritized values similarly, but preferred strikingly different management scenarios (Lam et al. 2019), suggesting different interpretations and contextualization of values, which included freedom. Divergent value interpretations were found also among four Indian fishing communities, which varied most in their prioritizations of freedom among the values investigated (Advani 2020). Ambiguity in interpretation and context of values and identities is often exploited politically. Also, differing interpretations of values by individuals and across contexts can yield fundamentally different beliefs and behaviors. Values are inherently good and thus vulnerable to co-option as truisms in rhetoric, as it is hard to refute someone who invokes a value to justify an action or decision. Despite their ambiguity, however, values have political salience, as seen by the ubiquitous polls of values administered around elections, referenda, and crises, including the current pandemic.

### Implicitness

Values and identities are typically implicit and only made explicit when invoked rhetorically for political purposes or revealed scientifically by empirical studies. In fisheries, stakeholders’ and communities’ values and identities are often assumed or espoused, but rarely investigated systematically. A prerequisite to responsive policies is the empirical elucidation of values. This has been done, for example, in food systems (Lam 2019b; Kaiser et al. 2021), fisheries (Song and Chuenpagdee 2015; Loring and Hinzman 2018; Johnson et al. 2019; Lam et al. 2019; Advani 2020), aquaculture (Bremer et al. 2016), ecological economics and sustainability science (Kenter 2016; Kenter et al. 2015, 2016, 2019), socio-technical systems (Pommeranz et al. 2012; Kaiser 2015), and environmental management (Gregory 2002; Gregory et al. 2012). In identity politics (Fukuyama 2018), social and political identities may be invoked explicitly to send politicized messages that impose certain values or conceptual framings to marginalize, stigmatize, or discriminate against specific groups, such as ethnic or indigenous minorities. Minorities also often invoke values and identities to rally solidarity and sympathy in political causes. Whether by in- or out-groups, values and identities can be used in political rhetoric, but without scientific underpinning. The implicit or explicit use of values and identities in the political sphere can obfuscate or incite political interests, respectively. More conceptual and empirical research into values and identities in the policy context thus is needed so that policies can be grounded in rigorous scientific and ethical analysis.

The plural, fluid and dynamic, ambiguous, and implicit natures of values and identities have hampered their explicit and nuanced consideration in fisheries management and policy. This has given fishery managers and policy-makers the freedom to make decisions largely independent of stakeholders’ and citizens’ values and identities. Making them explicit can hold decision-makers accountable. In particular, making the values and identities of fishery stakeholders and citizens transparent can aid decision-makers in designing policies that are more responsive to those affected by them. This can help to reconcile conflicts and resolve trade-offs at the science-society-policy nexus to sustainably manage marine resources. But an empirical science of values and identities in fisheries has lagged behind this practical stimulus. Fishery allocations are negotiated in the political arena, whereas fishery science tends to focus more on fish populations and biomasses than the human dimensions of fisheries. The COVID-19 pandemic instantiates an opportunity to rectify this knowledge gap, by exposing diverse values and identities of global seafood actors and their differential vulnerabilities and policy impacts, within and across scales and contexts.

## Differential vulnerabilities and impacts of COVID-19 on seafood actors at diverse scales and contexts

These general characteristics of values and identities make them notoriously hard to define and specify, let alone measure, so rather than adopt a static categorization of value or identity types (e.g., Schwartz 1992, 1994; Graham et al. 2013), I introduce a hierarchical conceptualization to describe seafood actors interacting within complex value chains and being affected by seafood and relevant policies implemented at diverse scales of human organization. At the level of the individual, actors make decisions based on their values and identities and policy-makers need to recognize their individuality, particularly their basic human dignity (Fukuyama 2018). At the social or community level, individuals may share social values and identities, so some fisheries and seafood policies can be more effective by targeting the shared values and identities of groups of individuals. Finally, at the cultural or societal level, individuals and social groups coalesce into collectives that can be described more saliently by broad characteristics, such as nationality and ethnicity, representing an aggregated set of shared values, beliefs, customs, and history. Key in this hierarchical conceptualization is to identify the appropriate scale of values and/or identities with political salience in the specific fishery, policy or governance context.

The three examples below illustrate the importance of scale and context, as exposed by the COVID-19 pandemic, in designing effective seafood governance strategies. Values and identities salient in policy and politics (e.g., Hornung et al. 2019; Lam 2021) can emerge at different scales of seafood value chains, as exemplified by: individual values and identities in local supply chains (India); social or communal values and identities in domestic (regional/national) supply chains (Canada); and cultural or societal values and identities in global supply chains (New Zealand). These examples highlight both the adverse consequences of a lack of differentiation and engagement at the appropriate scale of seafood actors in policy interventions (India and New Zealand), as well as the beneficial consequences of scale-differentiated actions by the industry (Canada). Effective seafood governance also must address multiple interlinked systems that call forth different values and identities in varied behavioral, social, economic, cultural, historical, political, and institutional spheres and contexts (Tsirogianni and Gaskell 2011; Kluckhohn and Strodtbeck 1961) . Human–environment interactions operate across multiple levels and scales and their dynamics are affected by institutions that similarly interact at multiple levels and scales (Cash et al. 2006). I conceptualize human values and identities hierarchically to help navigate the complex value and identity space and to position governance interventions at the appropriate scale so that they are fit for purpose. By recognizing the relevant scale of salient values and identities (Lam 2021), policy and governance solutions can be developed that are better tailored to their particular context (Young et al. 2018).

### India: individual values and identities in local supply chains

In India, the COVID-19 outbreak abruptly halted marine fisheries when the government imposed a national lockdown on March 24^th^, 2020 with just 4 h of notice (Balasubramanian 2020). The shock of the lockdown rippled through the nation’s economy and fishing-related livelihoods. Fishing was banned in most states until April 21^st^, after which it was declared an essential service (Purkait et al. 2020). India is the world’s second largest fish-producing nation, accounting for 6.56% of global production. Its fisheries sector employs 14.5 million people (roughly 1% of its total population), who live mostly in rural coastal communities. The sector contributes 1.1% of India’s GDP (Purkait et al. 2020), valued at 6.7 billion USD in 2018–2019 (Balasubramanian 2020). The lockdown disrupted ports, landing sites, ice factories, processing plants, transportation facilities, and market services, affecting diverse fishworkers across India’s seafood value chains, including fish harvesters and farmers, vendors, ice breakers, processors, distributors, traders, exporters, retailers, and consumers (Purkait et al. 2020; Balasubramanian 2020).

Exacerbating the COVID-19 public health crisis in India, second only to the USA in number of infections (JHU 2021), its population depends heavily on fish for food, with over 60% of the populace consuming fish (Purkait et al. 2020). Given this threat to domestic food security, many state governments in the first lockdown phase allowed small-scale beach landing crafts to operate with two to three fishers per craft, provided they could satisfy national health safety conditions, such as physical distancing at sea and at fish landing sites. In the southwestern state of Kerala, the operation of mechanized port-based boats (e.g., trawlers, purse seiners, and ring seiners) was banned, while small-scale fishers could fish for sustenance and domestic food security, often reaping higher yields and lower operating costs as fish shoals moved inshore in the absence of industrial fleets. Harvested fish in India are normally auctioned at landing sites in highly interactive, dynamic, crowded markets (Nayak et al. 2020) and bought by local consumers for food or by purchasers who transport fish for sale or processing in near and distant markets (Kurien 2020). In Kerala, amidst COVID-19 restrictions, local institutions, fisherfolk, cooperative officials, state administration, police, youth, and elders collaborated in village-level initiatives, emerging new fish market norms of physical distancing, weight-standardized prices, and fixed first-sale prices negotiated by a multi-stakeholder committee (Nayak et al. 2020). The power dynamics within the local seafood value chains shifted with these innovations: fishers received greater price stability and predictability, while the influential middlemen auctioneers and buyers, who provide credit to fishers and thus gain privileged access to their products, resisted them (ibid.). After lockdown measures were eased in June 2020, COVID-19 spread rapidly through fishing villages, with many asymptomatic cases and individuals, including village leaders, reluctant to go to quarantine centers, where fish was not offered for lunch (John Kurien, personal communication). This local tragedy of potentially avoidable deaths in fishing villages highlights the critical importance of tailoring policies not just to the values and identities salient in a given context (public health and fear for safety during the COVID-19 pandemic), but also to the appropriate scale of values and identities (individual dietary habits of fisherfolk), if they are to induce the desired behaviors (go to quarantine centres). This recognition and differentiation of values and identities by policy-makers can enhance policy compliance and effectiveness, both in response to COVID-19 (Lam 2021) and in fisheries (Lam et al. 2019; Advani 2020).

Given the vital role of fish as a source of food and livelihoods in India, the impact of COVID-19 on the fisheries sector has affected its entire economy and society. Understanding the values and identities of the diverse fisherfolk and their culture can help mitigate its impacts. Kurien (2020) has proposed a post-lockdown fishery development strategy consisting of a decentralized, modernized small-scale fishery supported by skilled human labor and knowledge-intensive technologies that respect “public health, decent work, ecological sanctity, energy efficiency, economic viability, employment opportunity, self-reliance, nutritional safety and equity.” He proposes that fish buyers be registered and obey rules of physical distancing, hygienic fish handling, food safety standards, and clean and proper storage, transportation and selling facilities, while fish sellers be issued quotas to sell specific fish species, be organized into collectives, and be linked to consumers to enhance their incomes and dignity (ibid.). This fishery development strategy resonates with an ethical harm principle proposed by Lam (2012), where fisheries are reorganized along different value priorities to minimize the environmental and social harm that they cause. In Lam’s proposal (ibid.), highly industrialized fishing enterprises that cause ecological damage would be “handicapped” financially relative to small-scale fisheries to reduce their economic efficiency in favor of social efficiency in marine resource extraction. Modernizing India’s small-scale fisheries similarly would prioritize social and energy efficiency and domestic food security (i.e., non-market social, environmental, and societal values) in local value chains over the economic efficiency (i.e., market value) of large-scale fleets that export fish commodities to feed international markets (Kurien 2020). These two proposals would foster not only sustainable, but also ethical development in fisheries.

### Canada: social values and identities in domestic supply chains

**Fig. 1:**
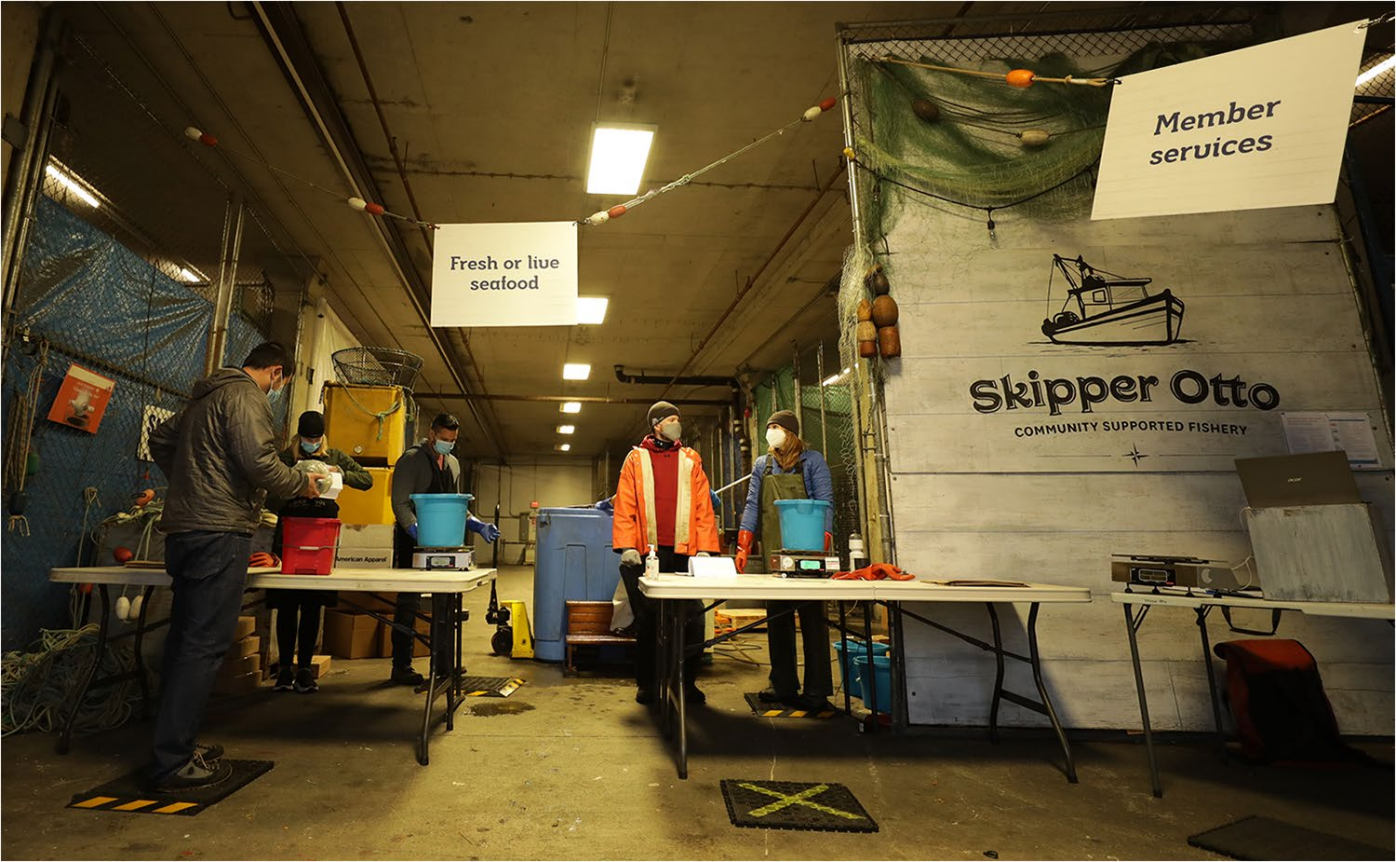
Operating under COVID-19 pandemic social lockdown conditions in November 2020, seafood pick-up location is open for Skipper Otto Community Supported Fishery (CSF) members at Fisherman’s Wharf, Vancouver, British Columbia, Canada (photo courtesy: Skipper Otto CSF)

In Canada, fisheries were declared an essential food-provisioning service at the outset of the COVID-19 crisis, but the ability of the industry to respond to the pandemic conditions has varied. One seafood business, Skipper Otto Community Supported Fishery (CSF; skipperotto.com), based in the western province of British Columbia (BC), has thrived with increased internet demand. Its business model is founded on transparent, fair, and sustainable regional and national supply chains. An annual membership fee gives members the opportunity to purchase online locally sourced, traceable, high-quality seafood available for pick up or delivery at multiple locations across the country (Fig. 1). Before the start of each fishing season, members buy a share of the catch, which they choose based on seafood type and quantity and can update during the season. With COVID-19, demand for direct fish sales via the internet increased dramatically and Skipper Otto’s memberships, volumes, and revenues surged (Sonia Strobel, personal communication). Despite the COVID-19 restrictions to social mobility and transport, Skipper Otto CSF was well-positioned to supply the seafood needs of existing and new members domestically, owing to its visionary business model, which also includes flexible supply chains, online presence, infrastructure, and established relationships (see also Stoll et al. 2021). Skipper Otto’s members not only gain access to better quality products and ensure fair prices to fish harvesters, but also indirectly commit to an environmental and social cause (skipperotto.com), which likely struck a chord of solidarity during the pandemic: “supporting Canadian fishing families through a revolutionary seafood buying experience.” Skipper Otto CSF is founded on three core values: “justice, equity, and pioneering creativity.” Its member community, composed of “fishing families, seafood lovers, and change-makers,” literally buy into its social values through their seafood purchases. Identifying with and sharing in Skipper Otto’s vision and values, its predominantly urban members engage in fisheries and social issues, such as contemporary racial justice and indigenous issues, via online newsletters and fora. Skipper Otto CSF thus explicitly infuses social values and identities in regional and national supply chains by labeling what, where, and how the seafood was caught to evolve more sustainable and fair practices, and thereby is promoting ethical development in their domestic seafood value chains.

In sharp contrast to Skipper Otto CSF, most industrial fisheries in Canada (and elsewhere) operate predominantly within global seafood supply chains. Heavy reliance on global seafood markets with mostly private corporate and investor control of fishing licences and catch quotas reduces resilience and flexibility within BC’s industrial fishery supply chain (Ecotrust 2020). With large fish export inventories and international market prices as key driving factors, investors and offshore buyers, not fish harvesters, largely control fishing access and market prices, leaving many local fishers disenfranchised, without autonomy to decide when or what to fish in response to the emergent pandemic conditions (ibid.). Counterbalancing this global market-driven situation within the industry, some fish harvesters, fishing industry organizations, non-profit organizations, and indigenous leaders forged an alliance to develop protocols for community-fisheries interactions to stabilize the fisheries market and to offer financial relief for local fishers. These local, multistakeholder collaborations, as the social initiatives in Kerala, India, are etching a new norm in ethical development, akin to the CSF model, of a more fair, resilient, localized fishing economy (ibid.) that supports local communities and domestic seafood value chains.

Even well-managed fisheries, such as the highly consolidated, vertically integrated, industrial-scale bottom-trawl groundfish fisheries in BC, were vulnerable to the emergent pandemic conditions. The fishing industry collaborated with a local ENGO in 2012 to reduce unwanted bycatch, defined as damage to the three-dimensional fish refuge habitat formed by sensitive sponge reefs and corals (Wallace 2007). This resulted in a complex and unique spatial bycatch agreement (Wallace et al. 2015) with the world’s first habitat “disincentive” quota. It used data from the complex multi-species quota management system and the 100% at-sea observer program (paid by the industry and in place since 1996) to establish a Habitat Conservation Bycatch Limit, which limited coral and sponge bycatch by individual vessels and the industrial fleet. Effective, accurate, and timely independent certified observers on fishing vessels are critical to manage these integrated Pacific groundfish trawl fisheries, which target sablefish, halibut, rockfish, hake, and other species (Thomson 2020). At-sea observers are responsible for on-board scientific data collection and monitoring of fishing activities and industry compliance with fishing regulations and licence conditions (Bender 2020). These are combined with dockside monitors to give a double third-party verification, which along with (self-reported) fisheries logs, give a tightly controlled fleet surveillance (Scott Wallace, personal communication). To reduce the public health risk posed by COVID-19, the federal regulatory body, Fisheries and Oceans Canada (DFO), abruptly suspended the at-sea observer program on 2 April 2020 (Gall 2020a). Prompted by the industry, on 14 April, DFO implemented an emergency electronic monitoring program with strategically positioned cameras and sensors to capture information on fishing activity, species identity, fish caught and discarded, and compliance with fishing regulations (Gall 2020b). This industry-led program helps to ensure a level playing field among fishing vessels by reducing illegal bycatch, discards, and other illegal, unregulated, and unreported (IUU) fishing typical of supertrawlers.

On the east coast of Canada, in contrast, to curb the expansion of supertrawlers and to protect the small-scale fleet, licence owners in the lobster fishery are required to operate their vessels, regulating against investor control of licences and quota. The maritime province of Nova Scotia accounts for half of the lobster exports from Canada, the world’s largest supplier of lobster, valued at 1.05 billion per year (Levinson-King 2020). This lucrative global fishery was not immune to the pandemic, however, being marred by long-standing cultural identity conflicts between indigenous Mi’kmaq and non-indigenous commercial lobster fishers (Slaughter 2020).

### New Zealand: cultural values and identities in global supply chains

In New Zealand, the COVID-19 pandemic created a shortage of migrant fishworkers for its deep-sea hoki fisheries, which supply lucrative global value chains. Previously, the plight of its migrant fishing crews was exposed by the revelation of forced and bonded labor aboard South Korean foreign charter vessels (Simmons and Stringer 2014). Such human rights violations in the global fishing industry often co-occur with reduced catch (Decker Sparks and Hasche 2019) and other illegal fishing practices, such as discharging bilge oil, high-grading quota species, and dumping rubbish and non-quota species (Simmons and Stringer 2014). The New Zealand government responded to the public outcry by requiring all foreign vessels fishing in its territorial waters to reflag to New Zealand, giving it full legal jurisdiction to require that they comply with its maritime rules, as well as workers’ qualifications and workers’ employment, health, and safety conditions (Kirk 2016). This national level response to the human rights violations was designed to ensure safe conditions and fair standards for all its fishing crews, both domestic and foreign (ibid.).

In the early stages of the pandemic, New Zealand’s strict lockdown requirements excluded entry of all foreigners, but were later relaxed to allow foreigners who provided essential services, including trained overseas fishing crews. As context, New Zealand’s fishing industry catches fish worth over 650 million a year, with exports worth over 1.5 billion a year (Kirk 2016). It also stimulates New Zealand’s economy and land-based workers through businesses that support fishing boats, such as catering firms, pharmaceuticals, and engineers (O’Connell 2020). New Zealand’s fishing industry relies heavily on migrant fishing crews with specialized qualifications, skills, and experience (Mohanlall 2020). During the pandemic, foreign crew workers have been required to self-isolate for 14 days in their home countries and to produce a negative COVID-19 test result prior to boarding flights to enter New Zealand. Upon entry, they must quarantine for 2 weeks in a government-run hotel and take another test on day 3. In one highly documented case in October 2020, *Sealord*, a Maōri-owned, transnational, vertically integrated fishing company, paid for a chartered flight of 235 Russian and Ukrainian skilled fish workers to enter the country, 18 of whom tested positive for COVID-19 on day 3 (Graham-McLay 2020). This outbreak caused a spike in the country’s infections and was followed by a second flight of 190 Russian mariners (Gate 2021), of whom 11 tested positive while in quarantine. The two flights and hotel stays cost approximately $1.2 million, paid by the fishing companies *Sealord* and *Independent Fisheries*.

This COVID-19 industrial fisheries example highlights the importance of recognizing the salient cultural values and identities of actors at various nodes in global fish chains, viz*.* the nationality of otherwise invisible fishing crew workers, as well as their vulnerabilities and impacts on other actors in the fishing and related business sectors. The foreign crew workers came predominantly from Russia, a country at the time with the fourth highest COVID-19 infection rate (JHU 2021), which made their identities relevant and visible to New Zealanders, even to citizens who do not profit from or consume fishery products. The interconnectedness of the global community through the market economy, transportation, and the current public health crisis forces both policy-makers and citizens not only to recognize the need for, but also to push for regulations that mandate safe work conditions for all essential workers, both domestic and foreign, as well as safe handling within global food value chains, as is being implemented in China (Henry Han, personal communication).

These three COVID-19 fishery examples illustrate the importance of recognizing the different scales at which salient values and identities can emerge in a given context: individual values and identities (such as individual dietary habits of local villagers within fishing communities) in local supply chains in India; social values and identities (such as pro-environmental and prosocial behavior of fish harvesters, purchasers, and consumers) in domestic supply chains in Canada; and cultural values and identities (such as national identities of foreign fishing crew members) in global supply chains in New Zealand. My hierarchical conceptualization of values and identities focuses on their multiplicity of scales to complement the identification and prioritization of specific values and identities in a given context (e.g., Lam et al. 2019; Advani 2020). It exposes the poor fitness-for-purpose if a policy or governance intervention is not matched to the salient scale in a given context, even if specific values and identities are adequately identified at a different scale. In the public health crisis instantiated by the global COVID-19 pandemic, national governments implemented categorical policy restrictions that did not account for different COVID-19 behavioral identities, which often led to low compliance and unintended health and socio-economic consequences for vulnerable minority groups (Lam 2021). Similarly, in fisheries, by not recognizing that salient values and identities emerge at different scales, decision-makers can implement policy and governance interventions with low compliance or unintended adverse consequences.

## Ethical governance of global seafood systems

The above three contextual examples illustrate how the global COVID-19 pandemic has impacted the global seafood industry, value chains, and fishworkers in varied and nuanced ways. Diverse seafood industry responses to the pandemic highlight both the vulnerability and the resilience of global seafood systems to pandemic-induced social mobility restrictions, disrupted transport and commodity flows, and shifts in consumer demands. In global supply chains, for example, limited availability of at-sea observers in the industrial Canadian groundfish trawl fisheries and of foreign crew in the New Zealand deep-sea fishery worsened monitoring, surveillance, and compliance, IUU fishing, and fish commodity flows. Meanwhile, the small-scale and community-supported (and industrial groundfish trawl) fisheries examples in India and Canada, respectively, illustrated the flexibility, responsiveness, and resilience of local fishworkers and organizations to design creative solutions (with social and technological innovations), to collaborate, and to develop value-based or ethical principles and norms of transparent, fair, and sustainable practices. Documented pandemic impacts to small-scale fisheries (Bennett et al. 2020) have been both adverse (e.g., increased health risks, further marginalization, and exacerbated vulnerabilities) and beneficial (e.g., revival of local food networks, increased local sales, and collective actions safeguarding rights). In the three examples here, the pandemic shock was met differentially, both by adaptive responses and inability to cope by diverse individuals, sectors, and fisheries, depending often on idiosyncratic conditions. Differential vulnerabilities and resilience across global seafood actors and value chains to COVID-19 have exacerbated disparities across fishworkers and fishing communities, while at the same time, have showcased some of the transformative possibilities for not only more sustainable, but also more ethical seafood governance that recognizes and responds to plural marine values and identities.

Collectively, these examples illustrate the importance of both context and scale in responding to emerging salient values and identities when designing policy interventions in times of shock. COVID-19 social and mobility restrictions immobilized many fishworkers, fishing vessels and transport ships, reduced access of seafood suppliers to markets, and disconnected many seafood actors along diverse value chains. These disruptions favored shorter, more local supply chains, and vertically integrated, industrial-scale operations able to move seafood seamlessly from producers to retailers. The Canadian domestic and global value chain examples illustrate these extremes: the community-supported fishery with direct online sales to consumers and the industrial groundfish trawl fisheries supplying the global market. Seafood demand has decreased significantly during the pandemic, with closures of restaurants, cafés, and hotels, which has affected not only the volume and price, but also the types of seafood traded (FAO 2020). The fresh seafood market dropped notably, with restaurants their major purchasers, while processed (notably frozen and canned) seafood sales have gone up, as consumers cook more at home (ibid). This increase in sales has been experienced, for example, by Skipper Otto CSF, whose business model previously catered to a niche market, but now with the COVID-19 pandemic, sells processed products to a more socially aware, mainstream market.

Additionally, these COVID-19 pandemic examples highlight the global seafood industry as a vital source of diverse values, including food, livelihoods, culture, and income (Lam 2019b). Multiple global seafood actors with often plural, fluid and dynamic, ambiguous, and implicit values and identities are interacting within diverse seafood value chains (Lam 2016), themselves interacting with complex food, economic, political, environmental, societal, and health systems (Kaiser et al. 2021) that represent multiple contexts and behavioral spheres (Tsirogianni and Gaskell 2011). Scale is stressed here to illustrate the salience of individual, social, and cultural values and identities within local, domestic (regional and national), and global value chains, respectively. COVID-19 policies designed to curb the global public health crisis are rippling through the global seafood industry via differential impacts on seafood actors, which indirectly are affecting a multitude of other interacting actors, institutions, and complex systems. Their reverberations in the global seafood industry are being experienced at multiple levels, for example, on village leadership in India, community cohesion in Canada, the national economies of fishing nations, and the global food system, as evidenced by the New Zealand deep-sea hoki fisheries. Thus, decision-makers in public health and natural resources need to consider explicitly the values and identities of affected stakeholders and citizens to enhance policy compliance and governance effectiveness, to foster ethical outcomes, and to reduce unintended consequences. This can be accomplished by decisions that prioritize focused on not only sustainable development, but also ethical development.

Embedding marine resource problems within a coherent ethical and scientific decision-analysis framework can enhance policy and governance outcomes. The often plural, fluid and dynamic, ambiguous, and implicit nature of values and identities makes fishery problems wicked (Jentoft and Chuenpagdee 2009; Lam et al. 2019) and post-normal (Lam et al. 2019; Kaiser et al. 2021), defying the definitive descriptions or unique solutions that characterize tame scientific problems (Rittel and Webber 1973). Diverse values, interests, and worldviews can clash over, for example, who owns or controls the resources, as seen in identity politics, and how to manage and allocate them, spawning value conflicts (Lam et al. 2019). At the science—society—policy nexus, value and identity conflicts can prevent the uptake of (potentially value-laden) scientific advice: facts and/or the legitimacy of knowledge producers or brokers may be disputed, depending on one’s values, which distorts policy outcomes by lack of buy-in, trust, and compliance. Integrating an ethical approach (see also Lam and Pauly 2010; Lam 2012, 2016; Lam and Pitcher 2012) with a scientific approach to marine sustainability problems would frame them inclusively, transparently, and deliberatively prior to proposing scientific methodologies or solutions. An ethical approach would make explicit the plural values and identities of seafood actors, as well as the differential vulnerabilities and potential management and policy impacts on affected stakeholders, citizens, and the environment. This ethical perspective would aid decision-makers to rationally analyze and resolve ethical dilemmas, value trade-offs, and resource conflicts.

Such an integrated ethical and scientific decision-analytic framework would foster more ethical governance, defined as participatory, deliberative, transparent, and accountable decision-making that seeks to synthesize diverse sources of knowledge and to reconcile the plural values and identities of affected stakeholders and citizens (modified from Lam et al. 2019). Ethical governance is composed of two essential components: deliberation and decision-support. As a precondition to making ethically informed decisions toward ethical food outcomes, Kaiser et al. (2021) propose that value-based deliberative processes (see also Dietz 2013) should be guided by procedural ethics with the following five key elements: (1) invite early all relevant food actors at the appropriate scale or level, (2) frame the problem as it appears in each constituency/region to reveal implicit values, (3) be politically non-partisan to seek common understandings and transparency, (4) invoke transdisciplinary science fit for societal purpose, and (5) adopt a post-normal science approach, as the facts are uncertain, values are disputed, the stakes are high, and decisions are urgent. To promote “ethical soundness” in ethical decision-making (Kaiser et al. 2007, p. 65), concrete ethical tools are needed that foster participatory deliberation and transparent decision-support through rational analysis.

Two ethical decision-support tools that have been applied to fisheries and seafood systems are the ethical matrix (Mepham 2000, 2008; FAO 2005; Kaiser et al. 2007; Lam and Pitcher 2012; Lam 2016) and the value- and ecosystem-based management approach (VEBMA; Lam et al. 2019). The ethical matrix (Mepham 2000, 2008) is a theoretically based conceptual tool that incorporates basic human interests as the ethical principles of wellbeing,^3^ autonomy, and justice (matrix columns) applied to different (human, animal, and environmental) interest or stakeholder groups (matrix rows). It combines the consequentialist (or utilitarian) and rights-based (or deontological) western ethics theoretical traditions, along with Rawls’ theory of “justice as fairness” (Rawls 1971). The matrix facilitates participatory rational ethical analysis and decision-making of seafood and fishery dilemmas by structuring factual and value-based assessments of the ethical impacts (and corresponding ethical duties) of proposed actions or policies on different affected stakeholders or groups (FAO 2005; Kaiser et al. 2007; Lam and Pitcher 2012; Lam 2016). Meanwhile, VEBMA makes transparent diverse stakeholder values and preferences underlying resource conflicts, in conjunction with the ecological and societal impacts and risks of alternative value-based management scenarios or policy choices (Lam et al. 2019). It thus evaluates the ecological viability, socioeconomic feasibility, and societal desirability of scenarios (ibid.). Both descriptive and evaluative, VEBMA culminates in a science-policy table that makes trade-offs explicit by qualitatively depicting, with a traffic-light coding, the socio-economic and ecological impacts and risks (table rows) for alternative value-based management scenarios or policy options (table columns) (ibid.). Such ethical tools prepare the ground for normative judgments based on ethical analysis and inclusive, transparent, and accountable decision-making. Ethical judgment involves diverse assessors (e.g., stakeholders, civil society, policy-makers, scientists, and NGOs) evaluating the impacts and risks on affected groups and then weighing the impacts and risks in terms of their societal acceptability in public policy decision-making.

## Conclusion

The COVID-19 pandemic has exposed problems in the sustainability and ethics of global seafood systems that impact not only the seafood actors within them, e.g., via workers’ conditions, social justice, and consumer choice, but also citizens globally, via, *inter alia*, food security, resource equity, and ecological integrity (Lam 2016). The global public health crisis has accentuated the relevance and complexity of designing policies tailored to the often plural, fluid and dynamic, ambiguous and implicit values and identities of affected stakeholders and citizens to influence their behavioral choices and to lessen adverse impacts (Lam 2021). Through crisis, the pandemic affords humanity an opportunity to rethink and to restructure global food systems (FAO 2020; Kaiser et al. 2021) toward more sustainable and ethical development and outcomes. Three COVID-19 seafood examples varying in geographical context (India, Canada, and New Zealand) and scale (local, domestic, and global value chains) illustrate the differential vulnerabilities of and policy impacts on diverse seafood actors. By explicitly recognizing the salient scale and context of seafood actors’ values and identities, decision-makers can craft nuanced policy responses and more effective governance solutions.

Just as the COVID-19 pandemic necessitates an ethical policy agenda (Lam 2021), so too does the global seafood industry, with three priorities: (1) to recognize the diversity of individuals, (2) to deliberate and negotiate value trade-offs, and (3) to promote public buy-in, trust, and compliance. Recognizing the diversity and basic dignity of seafood actors in marine resource management and policy can help overcome unsustainable practices and ineffective governance (see, e.g., Aguado et al. 2021). Deliberative processes that are inclusive and transparent (Kaiser et al. 2021) can avoid some of the identity politics (Fukuyama 2018) that often obfuscate management goals and lead to conflicts (Charles 1992; Lam et al. 2019). Complementing scientific approaches to environmental resource problems, concrete ethical deliberation and decision-support protocols and tools can promote rational analysis when evaluating trade-offs and making normative judgments to reconcile value and identity conflicts. Tailoring policies to the values and identities of diverse actors in seafood value chains (see, e.g., Advani 2020) will likely foster stakeholder buy-in, public trust, and regulatory compliance, which can enhance governance effectiveness. In sum, a coherent ethical and scientific decision-analytic framework can foster sustainable and ethical development through ethical governance by synthesizing diverse sources of knowledge and perspectives and reconciling the plural values and identities of affected stakeholders and citizens to resolve policy trade-offs and enhance societal resilience.

## Data Availability

Not applicable.

## Footnotes

1 This definition was developed with Matthias Kaiser in the Marie Skłodowska-Curie Individual Fellowship project, *Enhancing Seafood Ethics and Sustainability (eSEAS): A Values and Ecosystem-based Management Approach* (Lam 2019a), and refined by Lam (2019b).

2 European Commission Joint Research Centre *Science of Values and Identities in the Political Process* project: https://ec.europa.eu/jrc/en/enlightenment/call-experts-science-of-values. This Section draws heavily on a contribution by the author to the chapter, “What are useful case studies for the use of values and identities in politics?”, co-authored with Ana M. Jara and Mafalda Dâmaso.

3 The concept of wellbeing is a contraction of the principles of beneficence and non-maleficence in medical ethics (Beauchamp and Childress 2001).
